# A Peculiar Case of Neuralgia of the Trigeminus

**Published:** 1886-07

**Authors:** H. Hamecher


ARTICLE IX.

### A PECULIAR CASE OF NEURALGIA OF THE TRIGEMINUS,

BY H. HAMECHER, DENTIST, BERLIN.

In the first days of the month of January, a. c., Mrs. H., 52 years old, introduced herself to me, and asked me to remove the furious pains in the right side of her face, which pains, in her opinion, could only be caused by a diseased tooth. The anxious features of the lady, the timid expression of her eyes, the bowed down attitude, and her really woeful appearance exhibited a deep-seated suffering of long duration. Mrs. H. told me that she had been suffering from these vehement pains for the last five years, that these pains appeared periodically, that they had their origin in the gum of the suffering side, and then they radicated, with the rapidity of lightning, to the foramen infraorbitale, to the arcus zygomaticus and from there towards the temple. As the gum is very sensitive to the least pressure, and as the lightest touch provokes an attack, she, who had formerly been a remarkably corpulent lady, had for the last few years, lived exclusively on cacao and mucilaginous food; in fact, the patient presented the appearance of a walking skeleton. As the act of speaking for one part, was very difficult for Mrs. H., and for the other part, was anxiously avoided, from fear of provoking a new attack, I learned from her companion the following: Mrs. H. caught a severe cold five years ago, and since that time she suffered from these pains, which were not violent at the beginning, but gradually increased in severity. Four years and a half ago the pains had become so excessive that the patient, who was treated by physicians, without obtaining any relief, went to a quack by whom she was treated with the Baunsheid-method. By this the face became terribly swollen, indeed so badly that the family physician, during the treatment, entertained fears for the life of the patient. This treatment, too, was without any success, and Mrs. H., who considered her trouble incurable, was for a considerable length of time without any medical attendance. The suffering neither increased or decreased in severity. Then, suddenly, without any apparent cause, the pains would become so bad again, that Mrs. H. was compelled to look for medical attendance once more. The attending physician, after having given, for a long while, and in large doses, bromide of potassium, quinine, morphine, etc., declared the suffering a neuralgia of the trigeminus, for the cure of which an operation had to be performed. The patient, not feeling like submitting herself to that, applied to another physician, who gave her similar medicaments in another form. The last prescriptions, however, which were submitted to me for inspection did not contain bromide of potassium, etc., but solutio arsenicalis Fowleri. Mrs. H. took this solution of arsenic for many months, without experiencing the least relief. Like the nights of the last five years, the late ones have also been sleepless, even morphine had not the power of producing sleep. Outside of the fact, that the whole appearance of the patient made the diagnosis of a neuralgia of the trigeminus highly probable, the reputation of the physician, who had treated the case, seemed to me vouchsafing the correctness of the same. I accordingly declined to take any active part in the treatment, and intended to send the patient to a hospital, in order to have the neurotomy performed. But the entreaties of the sick lady were so imploring that I decided to examine her mouth.

All teeth had disappeared, the patient wearing an artificial set. To the right side of the upper jaw the roots of the incisors of the canine- and wisdom-teeth were perceptible. A pressure upon these roots did not provoke any pain, but the pains were felt as soon as the jaw was pressed in the region of the first and second bicuspids. At the request of the patient I extracted these roots, gave her internally morphine, and in large doses veratrine, to rub into the region of the temple. As was to be anticipated, my treatment met with almost no success whatever. The attacks appeared just the same as ever, but were moderated, of course by the veratrine. Finally, such an attack appeared on Friday, the 9th of January, and was so vehement that the patient came to me in despair and asked for help. With great decision she asserted that there was the root of a molar left in the jaw, from which the pains radiated. With her finger she pointed out the location of the praemolars.

But I could not discover anything which suggested the presence of a root in the gums. Certainly as soon as I touched the jaw, instantly the attack appeared. But this was the only symptoms that could objectively be established. The absorbed edges of the alveolus presented a little spot which felt slightly elevated. It was the well-known projection which is formed, by the healing process in case the alveolus is fractured during the extraction of a tooth. It was just this place, which was, as the patient said, the origin of her suffering, and the rigid upholding of this idea caused me to look for what might there be hidden. Of course, the operation could be executed only by having the patient completely under the influence of chloroform, which was established, not without apprehension, on account of the greatly reduced state of the patient's system. After having cut away the flesh of the jaw, there could be seen the regularly cicatrized edge of the alveolus. This was removed with a chisel, and, under this was imbedded the two-pronged root of the first bicuspid. I reseated the alveolus with a sharp forceps and removed the root. The nerve of the tooth, leading to the root, was pulled out of the alveolus in the length of about two centimetres, but did not show anything which could explain these violent attacks.

The patient recovered very rapidly. As soon as she became conscious, she laughed lively and positively asserted that now she was cured, respectively expected to be.

I saw the patient again on the 15th of January and on the 16th of February. Since the operation she sleeps during the nights, at first for several hours, and now normally; the pains decreased more and more.

At the last visit the patient told me her suffering was now so endurable that she had no desire for an improvement for the next fifty years to come.—*Dental Office and Laboratory.*

